# Cost-effectiveness analysis of COPD screening programs in primary care for high-risk patients in China

**DOI:** 10.1038/s41533-021-00233-z

**Published:** 2021-05-20

**Authors:** Shuli Qu, Xuedan You, Tianyi Liu, Lijiao Wang, Zheng Yin, Yanjun Liu, Chong Ye, Ting Yang, Mao Huang, Hongchao Li, Liwen Fang, Jinping Zheng

**Affiliations:** 1 Real-World Insights, IQVIA, Beijing, China; 2 Medical Affairs, AstraZeneca, Beijing, China; 3 National Clinical Research Center for Respiratory Diseases; Institute of Respiratory Medicine Chinese Academy of Medical Science; Department of Pulmonary and Critical Care Medicine, China-Japan Friendship Hospital, Beijing, China; 4 Department of Respiratory and Critical Care Medicine, The First Affiliated Hospital of Nanjing Medical University, Nanjing, China; 5 Department of Health Economics, School of International Pharmaceutical Business, China Pharmaceutical University, Nanjing, China; 6 National Center for Chronic and Non-Communicable Disease Control and Prevention, Chinese Center for Disease Control and Prevention, Beijing, China; 7 National Clinical Research Center for Respiratory Disease, The First Affiliated Hospital of Guangzhou Medical University, Guangzhou, China

**Keywords:** Population screening, Chronic obstructive pulmonary disease

## Abstract

We built a decision-analytic model to compare the cost-effectiveness of using portable spirometer and questionnaire to screen chronic obstructive pulmonary diseases (COPD) with no screening (i.e. usual care) among chronic bronchitis patient in China. A lifetime horizon and a payer perspective were adopted. Cost data of health services including spirometry screening and treatment costs covered both maintenance and exacerbation. The result indicated that portable spirometer screening was cost-saving compared with questionnaire screening and no screening, with an incremental cost-effectiveness ratio (ICER) of −5026 and −1766 per QALY, respectively. Sensitivity analyses confirmed the robustness of the results. In summary, portable spirometer screening is likely the optimal option for COPD screening among chronic bronchitis patients China.

## Introduction

Chronic obstructive pulmonary disease (COPD) refers to a progressive deterioration of lung function which may cause a series of mental and physical comorbidities. COPD is one of the top three causes of mortality worldwide and claimed 3.0 million lives in 2016^1^. The nation-wide study of COPD in China reported a prevalence of 8.2% among people aged over 40 in 2002–2004^2^. Other recent studies in 2018 reported a prevalence of 8.6% among people aged 20 or older^3^ and a prevalence of 13.6% among people aged 40 or order in China^4^. However, based on a study examining the disease-specific funding level and disease burden of China, compared with other leading causes of death such as ischemic heart disease, stroke, and diabetes, COPD received the least funding^5^.

People with early diagnosis have the chance to receive the COPD treatment earlier. The treatment of COPD can ameliorate the annual decline in the FEV1 and improve lung function and quality of life and resulted in a lower frequency of acute COPD exacerbations^6^. However, COPD patients frequently remain undiagnosed and untreated when the disease is in its mild forms^7^. A nationwide observational study in China reported that half of COPD patients were diagnosed when the disease was already in moderate-to-severe stage^4^.

Spirometry test is the diagnostic gold standard recommended by the Global Initiative for Chronic Obstructive Lung Disease (GOLD)^8^. It is a reliable and validated method for COPD diagnosis. However, considering the vast patient size and limited medical resources in China, the primary care clinicians may not have sufficient time to do the spirometry test for every patient. In addition, the purchasing price of the spirometry device is expensive, and may not be afforded by most primary care institutes. Therefore, the spirometry may not be an efficient and feasible tool in Chinese primary care situation^9^. Questionnaires for COPD screening, such as the COPD Diagnostic Questionnaire, are developed to reduce the cost and operational complexity^10^. The efficiency of peak expiratory flow (PEF) meters screening also has been validated by several studies in China and in other countries^11–15^. Questionnaires and portable spirometers for screening can be combined to increase the accuracy of COPD diagnosis in real-life community clinical practice^16^. Two systematic reviews concluded that all existing screening methods using either questionnaires alone or combined with portable PEF devices are beneficial compared with no screening^17,18^, i.e., they predict with at least 90% accuracy that the patient does not have COPD.

There is a public policy call to review the cost-effectiveness of COPD screening tests and to promote early diagnose of COPD in China. Thus, this study aimed to build a cost-effectiveness analysis (CEA) model to evaluate different COPD screening strategies among high-risk population from the healthcare system perspective. These results will fill an evidence gap and may be used to inform policy-making in COPD screening.

## Results

### Base case analysis

Results of the base case are shown in Table 1. Portable spirometer screening was cost-saving compared with questionnaire screening and no screening, with the incremental QALY of 0.05 and 0.37, and cost saving of ¥229 and ¥647, respectively. Portable spirometer is the optimal option for COPD screening among CB patients in China, and questionnaire is cost-saving option compared with no screening.

**Table 1:** Base case analysis results over a lifetime horizon.

Strategies	No screening/usual care (A)	Questionnaire screening (B)	Portable spirometer screening (C)
Costs (¥)	24,725	24,307	24,078
Life years	2.10	2.57	2.64
QALY	1.37	1.69	1.74
Incremental analysis	B vs. A	C vs. B	C vs. A
Δ Cost (¥)	−419	−229	−647
Δ QALY	0.32	0.05	0.37
ICER	−1,304 dominant	−5,026 dominant	−1,766 dominant

*QALY* Quality-adjusted life years; *ICER* Incremental cost-effectiveness ratio.

### Sensitivity analyses

**Fig. 1:**
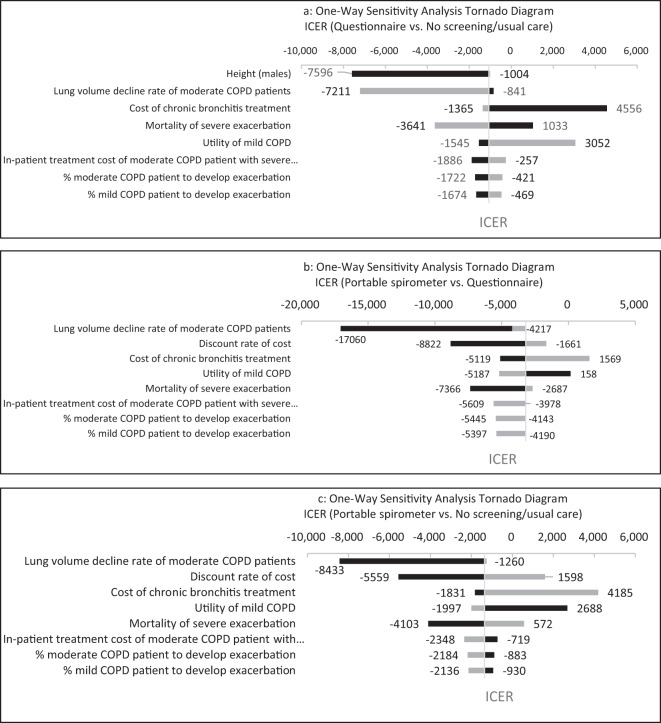
Tornado diagram of one-way sensitivity analysis. Light gray bar represented the lower limit, while dark gray line represented the higher limit of the parameter estimation.

The OWSA showed that the main drivers of the results of the three comparisons were height of male patients, lung volume decline rate of moderate COPD patients, and discount rate for costs. However, the results were robust to the parameter changes. Portable spirometer screening remained dominant treatment compared with questionnaire screening and no screening. Questionnaire screening has also remained dominant when compared with no screening (Fig. 1).

**Fig. 2:**
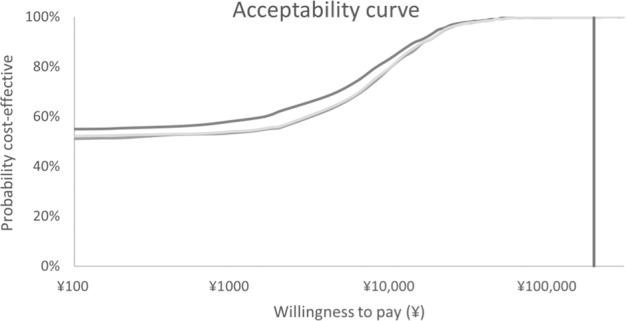
Acceptability curve. Cost-effectiveness acceptability curve in term of QALYs for Portable spirometer and questionnaire screening strategies versus no screening (usual care) in probability sensitivity analysis. Note: the *x*-axis was based on logarithm scale for better convenience to see.

The PSA showed that when WTP threshold set as ¥193,932 per QALY gained, the likelihood of portable spirometer screening being considered cost-effective were 100% compared with no screening and questionnaire screening (Fig. 2).

In scenario analysis, not performing a diagnostic pulmonary function spirometry test for those screening-positive patients in portable spirometer arm, portable spirometer produced a bigger cost-saving than in the base case, equal to ¥562 over 0.37 QALYs.

## Discussion

This study explored the cost-effectiveness of interventions aimed at identifying COPD patients among high-risk population in China. The result suggested that the portable spirometer can be a more promising tool for COPD screening in large scale for its high sensitivity and specificity compared with the screening questionnaire with low specificity. The OWSA results suggested that main drivers of uncertainty include height of male patients., lung volume decline rate of moderate COPD patients and discount rate. Height is associate with initial baseline values of FEV_1_ and lung function decline is associate with the disease progression and further impact the probability of exacerbation, pneumonia, and corresponding treatment costs. If COPD patients can be detected early and receive appropriate treatment at an early stage of COPD, the decline of their lung function can be ameliorated.

There are a few economic evaluations to compare the cost-effectiveness of different COPD screening strategies. We referred to the model structures in other published COPD-related CEA studies^19,20^ constructing this model. Thorn et al.^21^ suggested that the mini-spirometer could be an important device for pre-screening of COPD in primary care and may reduce the number of unnecessary spirometry tests performed. A cross-sectional study in India^22^ demonstrated that portable spirometers can help doctors detecting obstructive airways diseases with high sensitivity and specificity.

We utilized local data to make the model results more applicable to Chinese population. The normal range of predicted FEV_1_ formula was cited from a most recent nationwide study conducted in China^24^. The reliability and veracity of the reference equation has been verified by several studies^3,4^. The parameters of portable spirometer and screening cost were offered by its manufacturer. Local cost data and treatment pattern was collected from local literature or local KOL interview.

The values of parameters in base case were assumed to best represent practical clinical setting, and we also test scenario when not performing a diagnostic test for those screening-positive patients in portable spirometer arm. As the sensitivity and specificity of portable spirometer are accurate enough, it is controversial among clinical experts that whether a diagnostic pulmonary function spirometry test is still necessary. Therefore, we assumed the screening-positive patients to undergo a diagnostic test in the base case, but no diagnostic test in the scenario analysis. In this scenario, portable spirometer arm is still a dominant option.

**Fig. 3:**
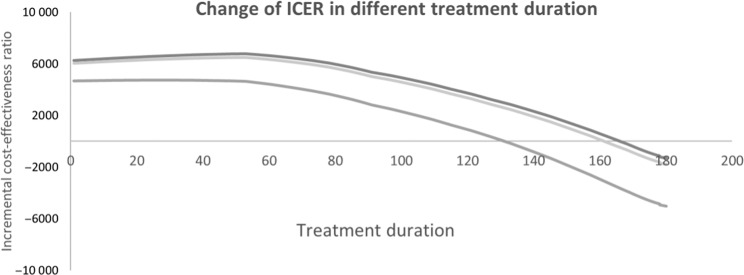
Change of ICER in different treatment duration. Note: the *x*-axis was based on logarithm scale for better convenience to see. Light gray line: portable spirometer vs. no screening (usual care); median gray line: questionnaire vs. no screening (usual care); and dark gray line: portable spirometer vs. questionnaire.

There was methodological controversy in constructing a CEA model for COPD screening. For example, Jordan et al.^25^ used a large RCT patient-level data to evaluate the cost-effectiveness of screening questionnaire, and suggested the systematic case-finding using screening questionnaire is cost-effective. However, Van Boven et al.^26^ argued that the 1-year study duration might not have been enough to support long-term economic assessment as COPD treatment is lifelong. In another study, Lambe et al.^23^ adopted a lifetime horizon, and evaluated systematic case-finding for COPD via modeling approach and received much more positive by Van Boven^27^. In our model, as the screening can detect COPD and those patients can receive appropriate treatment at early stage, the decline of their lung function can be ameliorated, and overall cost could be saved. Therefore, the longer we set the treatment duration, the lower we got the ICER from cost-effective to cost saving (Fig. 3).

Our study has two limitations: we used those utility scores reported in other countries due to a lack of China specific data. Sensitivity analyses suggested that the variation of input parameters only had a small impact on the ICERs; in addition, we did not consider other adverse events such as anemia and depression, and the impact on the pneumonia risk from ICS treatment, due to a lack of updated and relevant data.

In this study, we used CB patients as the targeted population. Potentially, the model can also be adapted to evaluate the cost-effectiveness in other COPD high-risk populations, such as smokers and emphysema patients, if the clinical and epidemiological data become available in future. In addition, the model can also be used to compare the cost-effectiveness of different types of portable spirometers. Both our study results and model can be used to inform policy-making in COPD screening in another high-risk population.

## Methods

### Screening population

Chronic bronchitis (CB) patients are at high-risk of COPD and can be diagnosed with COPD once persistent airway obstruction presents^28^. Patients with CB symptoms such as chronic cough, phlegm, and shortness of breath, especially during movement, had a nearly threefold increased risk of developing COPD compared with asymptomatic subjects^29^. The prevalence of CB is also higher in COPD patients^3,30^. Therefore, CB patients are main target populations for COPD screening and can be used as a good starting point to build a cost-effectiveness analysis model upon.

### Model structure

A decision-analytic model was constructed using Microsoft® Office Excel 2013 to simulate and evaluate the potential clinical and economic outcomes associated with two screening strategies of COPD: portable spirometer and questionnaire alone with no screening (i.e. usual care). The model has two parts: a decision tree model simulating the two screening processes and no screening process; a Markov model simulating the disease progression following the screening (using portable spirometer or questionnaire) and no screening process.

**Fig. 4:**
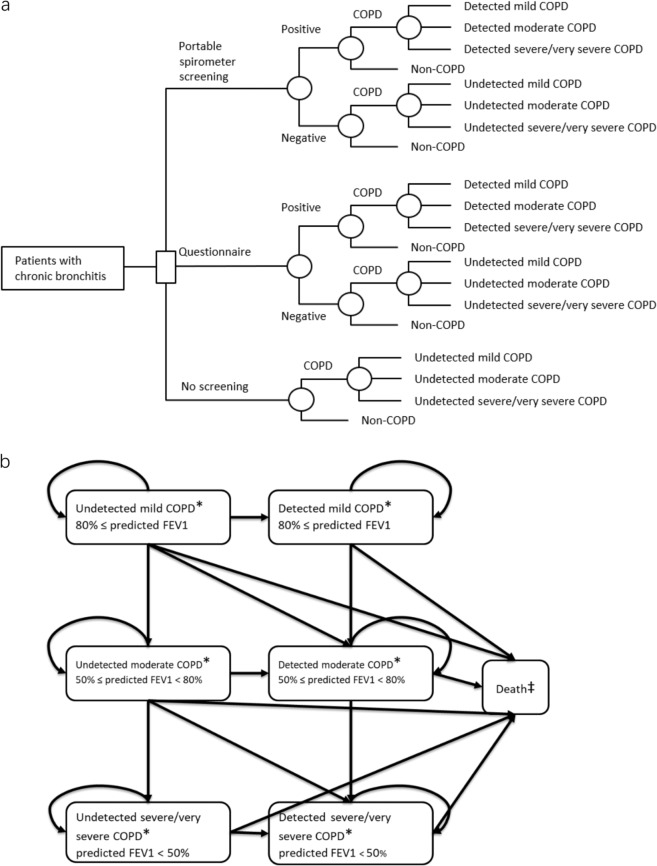
Cost-effective model structure. Model structure: Decision tree (a), Markov model (b). *Within each health state, patients may experience either severe exacerbation, non-severe exacerbation or serious pneumonia. ‡Death could occur following any health status.

Our decision tree model (Fig. 4a) assumed a one-time screening of 1000 Chinese CB patients in each of the three arms. Those 1000 CB patients may or may not have COPD. Sensitivity and specificity of the test were applied. Since screening tests may generate false positive results, we assumed that patients with a positive screening result will be referred to undergo diagnostic procedures. Patients with a false negative screening result (i.e. undetected COPD patients) will not be referred. After screening, those 1000 CB patients can be classified as non-COPD (remained as CB) patients, detected COPD patients or undetected COPD patients.

COPD is characterized by persistent respiratory symptoms and airflow limitation that is due to airway and/or alveolar abnormalities. The presence of a post-bronchodilator FEV1/FVC < 0.7 confirms the presence of persistent airflow limitation^8^. Regardless of diagnosis status, the severity of airflow limitation was classified according to the predicted post-bronchodilator FEV_1_ defined in GOLD 2020^8^ (mild, post-bronchodilator FEV_1_ >80% predicted; moderate, post-bronchodilator FEV_1_ ≥50% predicted; severe and very severe, post-bronchodilator FEV_1_ <50% predicted). Thus, at the end of the decision tree model, the simulated patients can be categorized into one of the following seven health states: Non-COPD, detected mild COPD, detected moderate COPD, detected severe/very severe COPD, undetected mild COPD, undetected moderate COPD, and undetected severe/very severe COPD.

The COPD patients will then enter into the corresponding states in the Markov model (Fig. 4b). A lifetime horizon and monthly cycle were applied. The undetected COPD patients can stay with original status, progress to the next severity status, become detected, or die. The detected can stay, progress to the next severity status, or die.

Undetected and detected COPD patients will receive different treatments, and consequently have different initial lung function variations. We assumed that undetected COPD patients would continue CB treatment (including inhaled corticosteriod, short-acting bronchodilation, expectorants, etc.), which is ineffective to improve lung function. Patients can have CB without COPD still need treatment advice and smoking cessation. Hence the FEV_1_ of undetected patients would decline. Detected COPD patients would initiate the standard COPD treatment. The lung function improvement benefited from COPD treatment was cited from the related clinical trials (Supplementary Table 6). Undetected patients might be detected while disease progressing.

Within each health state except for death, specific values for associated utility, costs, and risks of pneumonia and exacerbation were assigned. It was assumed that an exacerbation could be treated in hospital (severe) or in community care (non-severe), with different costs and dis-utilities. Severe exacerbation could be further classified into fatal or nonfatal. Pneumonia could also be fatal or nonfatal. Exacerbation and pneumonia were assumed to be independent events in the model^19^.

### Model inputs

The authors declare that the data analyzed during this study are available within the paper and its supplementary file.

#### Sensitivity and specificity of screening strategies

The sensitivity and specificity of the widely used portable spirometer e-LinkCare® PF 280 were provided by the manufacturer from an unpublished observational study in China. Spirometry was conducted and assessed in accordance with ATS/ERS recommendations^31,32^. Parameters for COPD screening questionnaires were cited from a meta-analysis^33^ (Table 2).

**Table 2:** Sensitivity and specificity of different screening approaches.

Parameter	Values (%)	Source/assumption	OWSA	
			Lower (%)	Upper (%)
Characteristics of questionnaire				
Sensitivity	87.50	^33^	78.75	96.25
Specificity	38.80	^33^	34.92	42.68
Characteristics of portable spirometer				
Sensitivity	99.90	e-LinkCare®	89.91	100.00
Specificity	97.70	e-LinkCare®	87.93	100.00

#### Baseline values of trough FEV1

COPD patients in different severity states had different initial baseline values of FEV_1_, which were used to calculate the FEV_1_ decline rate and transition probabilities between each severity state. Nationwide spirometric reference equation^24^ was applied in this study, and it fitted well to represent the normal range of predicted FEV_1_ for Chinese people (Eqs. 1 and 2).

1 $${\mathrm{FEV}}_{1{\mathrm{Male}}} = {\mathrm{exp}}[ - 10.61669 + 2.27078 \times {\mathrm{ln}}\left( {{\mathrm{height}}\,{\mathrm{in}}\,{\mathrm{cm}}} \right) + 0.06622 \times {\mathrm{ln}}\left( {{\mathrm{age}}\,{\mathrm{in}}\,{\mathrm{year}}} \right) + {\mathrm{Mspline}}]$$

2 $${\mathrm{FEV}}_{{\mathrm{1Female}}} = {\mathrm{exp}}[ - 9.69716 + 2.09385 \times {\mathrm{ln}}\left( {{\mathrm{height}}\,{\mathrm{in}}\,{\mathrm{cm}}} \right) + 0.02006 \times {\mathrm{ln}}\left( {{\mathrm{age}}\,{\mathrm{in}}\,{\mathrm{year}}} \right) + {\mathrm{Mspline}}]$$

The age and height of the screening cohort for each sex from published literature^3^ were applied to both equations (Table 3). The Mspline in the equations was referred to an age-specific contribution from the spline function. We used the midpoints of GOLD-criteria post-bronchodilator FEV_1_ thresholds for each health state: 90% for mild, 65% for moderate, and 25% for severe and very severe COPD states.

**Table 3:** Screening cohort characteristics.

Parameter	Values	Source/assumption	OWSA	
			Lower	Upper
Probability that a patient with chronic bronchitis (CB) has COPD	31.37%	^3^	25.10%	37.64%
Proportion of diagnosed COPD patients being mild	31.38%	^3^	25.10%	37.65%
Proportion of diagnosed COPD patients being moderate	46.04%	^3^	36.83%	55.25%
Proportion of diagnosed COPD patients being severe/very severe	22.58%	^3^	18.06%	27.10%
Average age of diagnosed COPD patients	59.59	^3^	47.67	71.50
% females in CB with COPD	23.46%	^3^	18.77%	28.15%
% females in CB without COPD	35.39%	^3^	28.31%	42.47%
Height (females)	155.80	^40^	140.22	171.38
Height (males)	167.10	^40^	150.39	183.81

#### Changes in trough FEV1 efficacy

The patients’ different COPD health states in the model were defined based on pulmonary function measured by the FEV_1_ percentage of predicted normal value, using the same severity classification as GOLD criteria. According to the GOLD classification of COPD severity of airflow limitation, the threshold for mild-to-moderate status was 80% of predicted FEV_1_, which were 1.77 L for females and 2.44 L for males, and the threshold for moderate-to-severe/very severe status was 50% of predicted FEV_1_, which were 1.11 L for females and 1.52 L for males.

Detected patients were assumed to receive inhaled treatment. Based on the treatment guideline and clinical practice in China, most mild patients receive mono bronchodilator such as SABA, SAMA, or LAMA; moderate patients receive LAMA or ICS + LABA; and severe/very severe patients receive LABA + LAMA, or ICS + LABA, or LABA + LAMA + ICS. The treatment effects of each therapy were obtained from several clinical trials conducted in Asian populations (Table 4).

**Table 4:** Clinical inputs.

Parameter	Values	Source/assumption	OWSA	
			Lower	Upper
COPD through FEV_1_ Decline (L/year)^a^	0.041	^41^	0.033	0.049
Annual probability to discover COPD from “undetected”^b^				
Mild COPD	20.00%	KOL interview	16.00%	24.00%
Moderate COPD	50.00%	KOL interview	40.00%	60.00%
Severe/very severe COPD	90.00%	KOL interview	72.00%	100.00%
Treatment effects (mean change in through FEV_1_ L/month)				
LAMA + LABA	0.026	Supplementary Table 3	0.021	0.031
LABA + ICS	0.031	Supplementary Table 3	0.025	0.037
LABA alone	0.013	Supplementary Table 3	0.010	0.016
LAMA alone	0.022	Supplementary Table 3	0.018	0.027
LABA + LAMA + ICS	0.025	Supplementary Table 3	0.020	0.030
% COPD patient to develop exacerbation				
Mild COPD	19.00%	^35,36^	15.20%	22.80%
Moderate COPD	19.00%	^35,36^	15.20%	22.80%
Severe/very severe COPD	26.50%	^35,36^	21.20%	31.80%
% of exacerbations treated in hospital				
Mild COPD	68.42%	^35,36^	54.74%	82.11%
Moderate COPD	68.42%	^35,36^	54.74%	82.11%
Severe/very severe COPD	66.04%	^35,36^	52.83%	79.25%
Mortality of severe exacerbation	1.28%	^37^	0.50%	10.00%
Risk of serious pneumonia				
Monthly incidence of serious pneumonia	0.20%	^38^	0.16%	0.25%
Mortality of serious pneumonia	3.33%	^38^	2.66%	3.99%

*SABA* *s*hort acting beta agonists, *SAMA* short acting muscarinic antagonists, *LAMA* long acting muscarinic antagonists, *LABA* long acting beta agonists, *ICS* inhaled corticosteroids, *KOL* key opinion leader.

^a^The FEV1 decline rate was transformed to L/month when used in the model. The transformed equation is: decline rate (L/month) = decline rate (L/year)/12.

^b^The annual detected probability was transformed to monthly probability when used in the model. The transformed equation is: monthly probability = 1−exp (ln (1-annual probability)/12).

Because of limited data on clinical efficacy, we assumed that the treatment effect was null after the first 6 months, and FEV_1_ declined since then. For undetected patients, they did not receive any treatment for COPD, thus their FEV_1_ kept decreasing from the first cycle. We cited different FEV_1_ decline rates for each COPD status^34^.

**Fig. 5:**
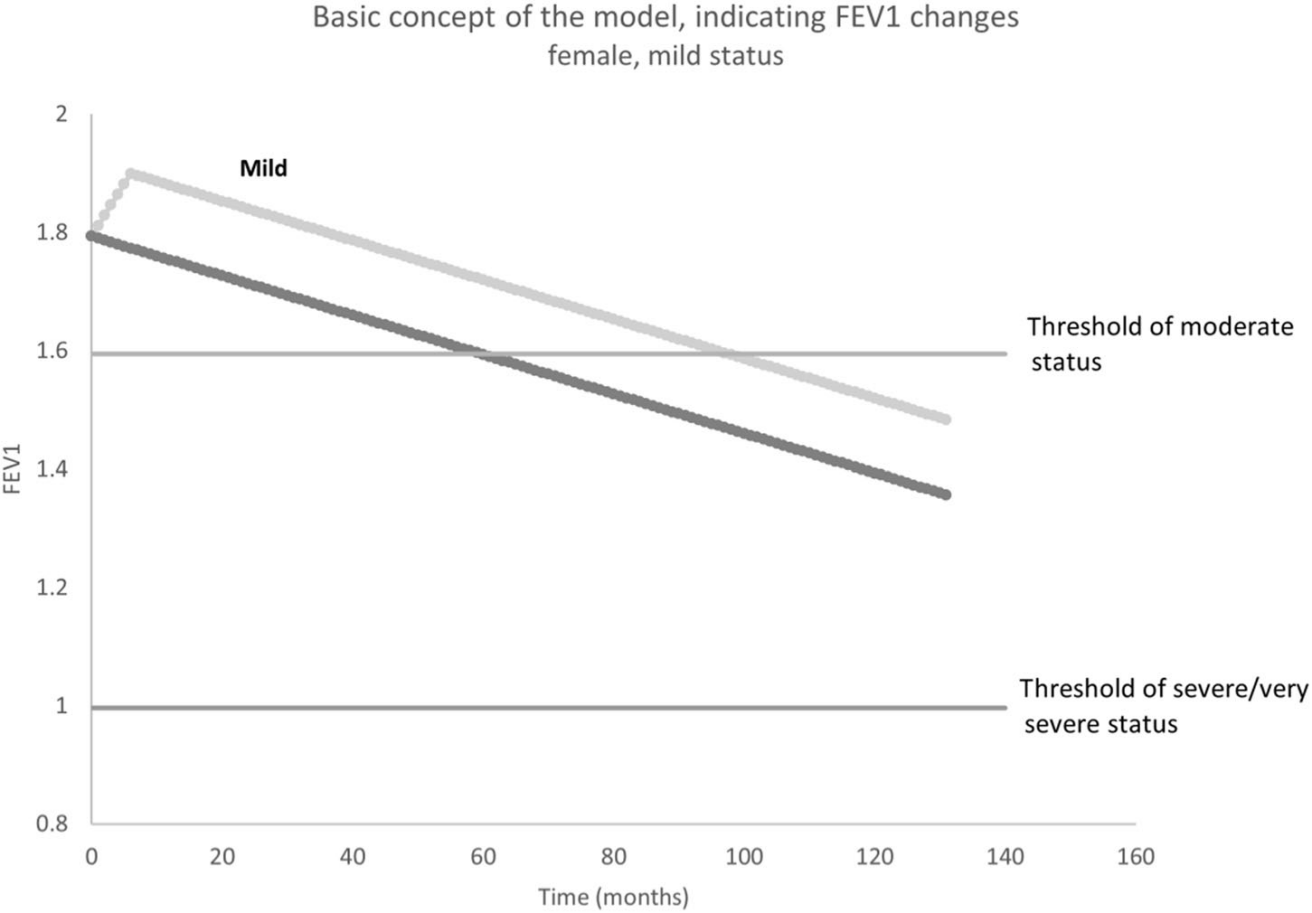
Illustration for FEV1 changing among females in mild COPD status. Light gray line represented the detected mild population, while dark gray line represented the undetected mild population.

Figure 5 illustrates the FEV_1_ changing process in mild COPD female patient in the model. For those undetected mild COPD patients, their FEV_1_ decline from the start point; however, the FEV_1_ of those detected patients would increase in the first 6 months and then decrease later on. Therefore, compared with undetected patients, it will take longer for detected patients to progress from mild to the moderate and severe/very severe status.

#### Exacerbation and pneumonia

Different severities of exacerbation and pneumonia are related with different mortality rates, costs and utilities (Table 5). Exacerbation and pneumonia risks were associated with the severity of airflow limitation and obtained from published studies^35–38^.

**Table 5:** Cost, utility, and discount rate input.

Parameter	Values	Source/assumption	OWSA	
			Lower	Upper
Drug costs per month (¥)				
LAMA + LABA	¥642	IQVIA CHPA 2018	¥513	¥770
LABA + ICS	¥114	IQVIA CHPA 2018	¥91	¥136
LABA alone	¥124	IQVIA CHPA 2018	¥99	¥149
LAMA alone	¥253	IQVIA CHPA 2018	¥202	¥303
LABA + LAMA + ICS	¥366	IQVIA CHPA 2018	¥292	¥439
Cost of COPD maintenance (oxygen inhalation, expectorant, etc) per month (¥)				
Mild COPD	¥23	^39^	¥18	¥27
Moderate COPD	¥67	^39^	¥54	¥81
Severe/very severe COPD	¥187	^39^	¥150	¥225
Cost of CB treatment per month (¥)	¥288	IQVIA CHPA 2018	¥100	¥500
Amoxicillin	¥10	IQVIA CHPA 2018	–	–
Dextromethorphan	¥27	IQVIA CHPA 2018	–	–
Ambroxol hydrochloride	¥224	IQVIA CHPA 2018	–	–
Aminophylline	¥20	IQVIA CHPA 2018	–	–
Ipratropium bromide	¥7	IQVIA CHPA 2018	–	–
AE costs (¥)				
Exacerbation treated in inpatient				
Mild COPD	¥8,639	^39^	¥6,911	¥10,366
Moderate COPD	¥17,277	^39^	¥13,822	¥20,732
Severe/very severe COPD	¥25,915	^39^	¥20,732	¥31,099
Exacerbation treated in outpatient	¥395	^39^	¥316	¥474
Cost of pneumonia	¥32,394	^39^	¥25,916	¥38,873
Screening and diagnosis costs (¥)				
Cost of portable spirometer screening	¥34	Supplementary Table 9	¥27	¥41
Cost of questionnaire screening	¥8	Supplementary Table 9	¥6	¥9
Cost of additional tests to confirm COPD	¥220	Supplementary Table 8	¥176	¥264
Utility				
Mild COPD	0.81	–	0.65	0.97
Moderate COPD	0.72	^20^	0.58	0.86
Severe/very severe COPD	0.67	^20^	0.54	0.80
Correction for exacerbation in outpatient	0.85	^36^	0.68	0.99
Correction for exacerbation in inpatient	0.50	^36^	0.40	0.60
Correction for serious pneumonia	0.50	^36^	0.40	0.60
Discount rate				
Effect	3.5%	^42^	0.00	5.00
Costs	3.5%	^42^	0.00	5.00

Exchange rate in 2018 was 1 Chinese Yuan = 0.1511 US Dollar.

*KOL* key opinion leader, *CB* chronic bronchitis, *CHPA* Chinese Hospital Pharmaceutical Audit database, *SABA* short acting beta agonists, *SAMA* short acting muscarinic antagonists, *LAMA* long acting muscarinic antagonists, *LABA* long acting beta agonists, *ICS* inhaled corticosteroids.

#### Costs

All costs included in the model were in Chinese Yuan and adjusted into 2018 price with CPI (consumer price index; Table 5).

The costs for screening programs and diagnosis tests were collected via key opinion leader (KOL) interviews. The costs of diagnosis tests included outpatient visit fee, cost of bronchodilators, and cost of lung function test and chest radiography.

The unit prices of each inhaled treatment for COPD were collected from IQVIA Chinese Hospital Pharmaceutical Audit Database (CHPA). The CHPA database captures purchase statistics from over 9000 hospitals in China and reports the market prices at which the panel hospitals purchase products from wholesalers, distributors, and manufacturers. For each inhaled treatment category, the weighted average unit price was calculated using total sales amount divided by sales volume. Then we calculated the monthly prescription costs using the weighted average unit price and the recommended dose of each treatment.

Other cost, including maintenance treatment (e.g. oxygen inhalation, expectorant, etc.), treatment costs of exacerbation and pneumonia were obtained from Fan^39^ and adjusted by KOLs.

Undetected COPD patients were assumed to chronically receive CB treatment (including anti-infection, antibechic, expectorants, and antispasmodic etc. ¥288 per month). Unit cost of CB treatment were collected from IQVIA CHPA database, and the dosage were obtained from the treatment guideline verified by clinical expert. Detailed cost component can be found in Supplementary Tables 4, 7, 8, and 9.

#### Utility

Utility values were derived from previously published literatures and related to disease severity, exacerbation and pneumonia (Table 5). These utility values were used to estimate quality-adjusted life-years (QALYs) by multiplying the number of accrued life years within each health status by the utility weight of each disease severity.

### Sensitivity analyses

One-way sensitivity analysis (OWSA) were performed to investigate the impact of variation in key model-input values individually, within reasonable fixed limits, on the base case incremental cost-effectiveness ratio (ICER). As suggested and confirmed by a panel of local clinical experts, the height of patients, sensitivity and specificity of screening strategies varied by ±10%, the risk of death with severe exacerbation (treated in hospital) ranged from 0.5% to 10%, and those of the other parameters varied by ±20% (Tables 2–5).

Probabilistic sensitivity analysis (PSA) was performed to test the effect of parameter uncertainty on the study results. Measures of distribution were obtained from the literature. We calculated the ICER by running 1000 Monte Carlo simulations to determine the proportions of simulations that were under predefined willingness-to-pay (WTP) thresholds of three times the gross domestic product (GDP) per capita in 2018. Then, a cost-effectiveness acceptability curve was generated to summarize the uncertainty of the cost-effectiveness analysis and determine the proportions of simulations that were under the WTP thresholds. The values and distributions of parameters for sensitivity analyses were shown in supplementary file.

Since the accuracy rate of portable spirometer is very high, we also tested the scenario that diagnostic test was not performed in those screening-positive patients in portable spirometer arm.

### Reporting summary

Further information on research design is available in the Nature Research Reporting Summary linked to this article.

## Supplementary information

Reporting Summary

Supplementary Information

## Data Availability

The data that support the findings of this study are available from the corresponding author upon reasonable request.
