# Supplementary material for: Cost-effectiveness analysis of COPD screening programs in primary care for high-risk patients in China
Source: NPJ Prim Care Respir Med. 2021 May 20;31:28. doi: 10.1038/s41533-021-00233-z (PMC8137942; doi:10.1038/s41533-021-00233-z)
Supplement: Supplementary file 2 — Supplementary Information [file 41533_2021_233_MOESM2_ESM.pdf]

Supplementary file

Supplementary Table 1. The values of sensitivity and specificity of different screening approaches and probabilities of discovering COPD during disease progression in probabilistic sensitivity analyses

Parameter	Values in base case	PSA
		Distribution (α, β) or (μ, sd)
Characteristics of questionnaire		
Sensitivity	87.50%	Beta (47.14, 6.73)
Specificity	38.80%	Beta (234.72, 370.23)
Characteristics of portable spirometer		
Sensitivity	99.90%	Beta (0.54, 0.054)
Specificity	97.70%	Beta (22.18, 0.52)

[1] the annual detected probability was transformed to monthly probability when used in the model. The transformed equation is: monthly probability = 1-exp (ln (1-annual probability)/12).

Supplementary Table 2. The values of screening cohort characteristics in probabilistic sensitivity analyses

Parameter	Values in base case	PSA
		Distribution (α, β) or (μ, sd)
Probability that a patient with chronic bronchitis (CB) has COPD	31.37%	Beta (65.60, 143.51)
Proportion of diagnosed COPD patients being mild	31.38%	Dirichlet (65.59, 143.44)
Proportion of diagnosed COPD patients being moderate	46.04%	Dirichlet (51.36, 60.19)
Proportion of diagnosed COPD patients being severe/very severe	22.58%	Dirichlet (74.13, 254.15)
Average age of diagnosed COPD patients	59.59	Normal (59.59, 6.08)
% females in CB with COPD	23.46%	Beta (73.27, 239.06)
% females in CB without COPD	35.39%	Beta (61.70, 112.65)
Height (females)	155.80	Normal (155.80, 7.95)
Height (males)	167.10	Normal (167.10, 8.53)

Supplementary Table 3. The values of clinical inputs of the Markov model in probabilistic sensitivity analyses

Parameter	Values in base case	PSA
		Distribution (α, β) or (μ, sd)
COPD FEV_1 Decline (L/year)[1]	0.041	Normal (0.041, 0.0042)
Annual probability to discover COPD from "undetected"‡		
Mild COPD	20.00%	Beta (76.63, 306.53)
Moderate COPD	50.00%	Beta (47.52, 47.52)
Severe/very severe COPD	90.00%	Beta (14.98, 1.66)
Treatment effects (mean L/month)		
LAMA+LABA	0.026	Normal (0.026, 0.0027)
LABA+ICS	0.031	Normal (0.031 0.0031)
LABA alone	0.013	Normal (0.013, 0.0013)
LAMA alone	0.022	Normal (0.022, 0.0023)
LABA+LAMA+ICS	0.025	Normal (0.025, 0.0025)
% COPD patient to develop exacerbation		
Mild COPD	19.00%	Beta (77.6, 330.83)
Moderate COPD	19.00%	Beta (77.6, 330.83)
Severe/very severe COPD	26.50%	Beta (70.32, 195.05)
Probability of hospitalization due to severe exacerbation		
Mild COPD	68.42%	Beta (29.64, 13.68)
Moderate COPD	68.42%	Beta (29.64, 13.68)
Severe/very severe COPD	66.04%	Beta (31.96, 16.44)
Mortality of severe exacerbation	1.28%	Beta (0.26, 20.17)
Risk of serious pneumonia		
Monthly incidence of serious pneumonia	0.20%	Beta (95.84, 46694.81)
Mortality of serious pneumonia	3.33%	Beta (92.81, 2696.17)

[1] the FEV_1 decline rate was transformed to L/month when used in the model. The transformed equation is: decline rate (L/month) = decline rate (L/year)/12.

SABA: short acting beta agonists; SAMA: short acting muscarinic antagonists; LAMA: long acting muscarinic antagonists; LABA: long acting beta agonists; ICS: inhaled corticosteroids

Supplementary Table 4. The values of cost input in probabilistic sensitivity analyses

Parameter (¥)	Values in base case	PSA
		Distribution (α, β) or (μ, sd)
Drug costs per month		
LAMA+LABA	¥642	Gamma (1, 641.77)
LABA+ICS	¥114	Gamma (1, 113.54)
LABA alone	¥124	Gamma (1, 124.03)
LAMA alone	¥253	Gamma (1, 252.66)
LABA+LAMA+ICS	¥366	Gamma (1, 366.20)
Cost of COPD maintenance treatment per month		
Mild COPD	¥23	Gamma (1, 22.67)
Moderate COPD	¥67	Gamma (1, 67.37)
Severe/very severe COPD	¥187	Gamma (1, 187.17)
Cost of chronic bronchitis treatment	¥275	Gamma (1, 275)
AE costs		
cost of exacerbation		
Treated in inpatient		
Mild COPD	¥8,639	Gamma (1, 8638.50)
Moderate COPD	¥17,277	Gamma (1, 17277.00)
Severe/very severe COPD	¥25,915	Gamma (1, 25915.50)
Treated in outpatient	¥395	Gamma (1, 394.72)
Cost of pneumonia	¥32,394	Gamma (1, 32394.38)
Screening and diagnosis costs		
Cost of portable spirometer screening	¥34	Gamma (1, 34.00)
Cost of questionnaire screening	¥8	Gamma (1, 7.50)
Cost of additional tests to confirm COPD	¥220	Gamma (1, 220.00)

KOL: key opinion leader; SABA: short acting beta agonists; SAMA: short acting muscarinic antagonists; LAMA: long acting muscarinic antagonists; LABA: long acting beta agonists; ICS: inhaled corticosteroids

Supplementary Table 5. The values of utility and discount rate in probabilistic sensitivity analyses

Parameter	Value in base case	PSA<br>Distribution (α, β) or (μ, sd)
Utility		
Mild COPD	0.81	Beta (17.44, 4.09)
Moderate COPD	0.72	Beta (26.17, 10.18)
Severe/very severe COPD	0.67	Beta (31.02, 15.28)
Correction for exacerbation in outpatient	0.85	Beta (13.56, 2.39)
Correction for exacerbation in inpatient	0.50	Beta (47.52, 47.52)
Correction for serious pneumonia	0.50	Beta (47.52, 47.52)
Discount Rate		
Effect	3.5%	Normal (0.035, 0.013)
Costs	3.5%	Normal (0.035, 0.013)

Supplementary Table 6. Reported FEV_1 improvement of COPD treatments

COPD treatment	Intervention	Mean (ml/weeks)	Mean (ml/month)	Mean (L/month)	Reference[1]
LAMA + LABA	GFF 18/9.6ug	165.000	29.874	0.030	[1]
	IndaGlyco 110/50ug	123.530	22.365	0.022	[2]
LABA + ICS	FFVI 100/25ug	180.000	32.589	0.033	[3]
	FFVI 50/25ug	140.000	25.347	0.025	[3]
	FFVI 200/25ug	190.000	34.400	0.034	[3]
	SFC 500 /twice a day	115.980	20.998	0.021	[2]
LABA alone	FF 9.6ug	72.000	13.036	0.013	[1]
LAMA alone	GP 18ug	59.000	10.682	0.011	[1]
	Glyco 50ug	137.000	24.804	0.025	[4]
	Tio 5ug	69.000	12.493	0.012	[5]
	Tio 18ug	230.000	41.642	0.042	[6]
LABA + LAMA + ICS	320/18/9·6 µg BGF MDI	147.000	26.615	0.027	[7]
	18/9·6 µg GFF MDI	125.000	22.631	0.023	[7]

[1] *All the trials were conducted in Asian population and lasted 24 weeks at least*

SABA: short acting beta agonists; SAMA: short acting muscarinic antagonists; LAMA: long acting muscarinic antagonists; LABA: long acting beta agonists; ICS: inhaled corticosteroids.

GFF: glycopyrrolate/formoterol fumarate; IndaGlyco: indacaterol/glycopyrronium; FFVI: fluticasone/vilanterol; SFC: fluticasone/salmeterol; FF: formorterol fumarate; GP: glycopyrrolate; Glyco: glycopyrronium; Tio: tiotropium; BGF: budesonide/glycopyrrolate/formoterol; MDI: metered-dose inhaler

Supplementary Table 7. Maintenance cost (per month) for each health state

Item	unit cost	Resource using times per patient per 3 months		
		mild	moderate	severe/very severe
Drug cost				
Influenza vaccination	¥83.98	0.25	0.25	0.25
Phlegm agent	¥3.375	0	39.74	64.455
Oxygen therapy	¥3.75	0	0	61.065
Medical service				
Clinican service/ per visit for respirary department	¥50	0.5	0.5	1
Lab examination				
Lung volume examination	¥44	0.5	0.5	1
Maintenance Cost per 3 months		¥67.995	¥202.12	¥561.52
Maintenance Cost per month		¥22.67	¥67.37	¥187.17

* cited from Fan 2016[8] and adjusted by local clinical experts

Supplementary Table 8. Diagnosis cost (in CNY)

Item	Cost
Outpatient fee	50
Bronchodilators	25
Lung function test	75
Chest X-ray	70
Total	220

* provided by local clinical experts

Supplementary Table 9. Screening cost (in CNY)

The average wage of clinicians in primary care is ¥60,000. Assumed 250 working days per annum and 8 working hours per day. Therefore, the average labor cost per minute is ¥60,000/(250*8*60)= ¥0.5.

	Questionnaire	Portable spirometer
Unit price	¥0	¥35,000
Service life year	-	3 years
Cost of consumable items	¥0	¥25
Time for screening	15 min	15 min
Labor cost per minute	¥0.5	¥0.5
Screening cost per patient		
Labor cost	15min*¥0.5/min=¥7.5	15min*¥0.5/min=¥7.5
Depreciation	0	¥35,000/(3*250*8*60/15)=¥1.5
Consumable items	0	¥25
Total	¥7.5	¥34

Reference

[1]Lipworth BJ, Collier DJ, Gon Y, Zhong N, Nishi K, Chen R, et al. Improved lung function and patient-reported outcomes with co-suspension delivery technology glycopyrrolate/formoterol fumarate metered dose inhaler in COPD: a randomized Phase III study conducted in Asia, Europe, and the USA. International journal of chronic obstructive pulmonary disease. 2018;13:2969.
[2]Zhong N, Wang C, Zhou X, Zhang N, Humphries M, Wang L, et al. Efficacy and safety of indacaterol/glycopyrronium (IND/GLY) versus salmeterol/fluticasone in Chinese patients with moderate-to-severe chronic obstructive pulmonary disease: the Chinese cohort from the LANTERN study. COPD: Journal of Chronic Obstructive Pulmonary Disease. 2016;13(6):686-92.
[3]Zheng J, de Guia T, Wang-Jairaj J, Newlands AH, Wang C, Crim C, et al. Efficacy and safety of fluticasone furoate/vilanterol (50/25 mcg; 100/25 mcg; 200/25 mcg) in Asian patients with chronic obstructive pulmonary disease: a randomized placebo-controlled trial. Current medical research and opinion. 2015;31(6):1191-200.
[4]Wang C, Sun T, Huang Y, Humphries M, Bai L, Li L, et al. Efficacy and safety of once-daily glycopyrronium in predominantly Chinese patients with moderate-to-severe chronic obstructive pulmonary disease: the GLOW7 study. International journal of chronic obstructive pulmonary disease. 2015;10:57.
[5]Ma J, Zhou Z, Tang Y, Zhong N. The efficacy and safety of low-dose tiotropium bromide inhaled via Respimat in patients with chronic obstructive pulmonary disease. Chinese Journal of Inernal Medicine. 2014;53(12):964-8.
[6]Wang T, Luo G, Hu Y, Li F, Ma J, Wang J, et al. Comparative study on the efficacy of tiotropium bromide inhalation and oral doxofylline treatment of moderate to severe stable chronic obstructive pulmonary disease. Journal of Huazhong University of Science and Technology [Medical Sciences]. 2011;31(5):614.
[7]Ferguson GT, Rabe KF, Martinez FJ, Fabbri LM, Wang C, Ichinose M, et al. Triple therapy with budesonide/glycopyrrolate/formoterol fumarate with co-suspension delivery technology versus dual therapies in chronic obstructive pulmonary disease (KRONOS): a double-blind, parallel-group, multicentre, phase 3 randomised controlled trial. The Lancet Respiratory Medicine. 2018;6(10):747-58.
[8]Fan C. The Cost Effectiveness Analysisof Indacaterolversus Tiotropiumin Chinese Medical Cost Setting. Drug Evaluation. 2016(2016.01):34-9.
